# The Psychological Impact of Isotretinoin Therapy on Acne Vulgaris Patients

**DOI:** 10.7759/cureus.50612

**Published:** 2023-12-16

**Authors:** Awadh Alamri, Randa Khafaji, Atheel Balkhy, Sahal Samarkandy, Ali Alraddadi

**Affiliations:** 1 Dermatology, King Abdulaziz Medical City, Jeddah, SAU; 2 Medicine and Surgery, King Abdullah Medical Complex, Jeddah, SAU; 3 Dermatology, Saudi Commission for Health Specialties, Jeddah, SAU

**Keywords:** anxiety, depression, psychological impact, isotretinoin, acne vulgaris

## Abstract

Introduction

Acne vulgaris is a dermatologic condition that affects the pilosebaceous unit. It is the most common skin disorder worldwide, and it is most prevalent during puberty. For patients with moderate to severe acne that is unresponsive to antibiotic treatment, the main treatment is oral isotretinoin. The objective of this study was to define the impact of isotretinoin and its well-established adverse effects on the psychological status of acne patients during treatment.

Methods

The sample cohort included 80 patients with acne vulgaris who were treated with isotretinoin. The sample size was calculated using the Raosoft [Raosoft.com (2015) Sample Size Calculator by Raosoft, Inc.] sample size calculator to maintain a confidence interval of 95% and a margin of error of 5%. The participants were assessed using the Depression and Anxiety Stress Scale-21 (DASS-21). The DASS-21 is a self-reporting scale used to measure the emotional states of depression, anxiety, and stress. This study was a cross-sectional survey conducted at the dermatology outpatient clinic at King Abdulaziz Medical City, Jeddah, Saudi Arabia. All patients diagnosed with acne vulgaris between January and April 2020 were included in the study. A multivariate regression analysis was also conducted to determine the presence of any significant independent factors associated with depression, anxiety, and stress. A P-value of ≤0.05 was considered statistically significant.

Results

We observed that the most prevalent psychiatric disorder among acne patients on isotretinoin therapy was anxiety. Our results also indicate that a history of mental illness is an independent risk factor for developing depression when isotretinoin is used. In addition, known adverse physical effects of isotretinoin treatment, including muscle pain, arthralgia, and headaches significantly increase the likelihood of patients developing psychiatric morbidity during therapy.

Conclusion

Isotretinoin is a highly potent therapy for acne. Overall, the physical side effects profile is well acknowledged, yet the exact psychological impact the treatment predisposes the patients to is yet to be determined.

## Introduction

Acne vulgaris is a dermatologic condition that affects the pilosebaceous unit. Obstruction and inflammation of the pilosebaceous glands leads to the formation of comedones, papules, pustules, nodules, and cysts and may also lead to scarring [1]. Acne is the most common skin disorder worldwide, with a higher prevalence during adolescence, since it peaks during puberty. It is estimated that acne affects approximately 85% of individuals between the ages of 12 and 25, as documented in the Global Burden of Disease study [2]. Acne is classified into three main subcategories according to its severity. These are, a) mild acne - presenting as comedones and possibly a few papulopustules; b) moderate acne - which presents with a greater number of papules and pustules; and c) severe nodulocystic acne - which presents with nodules >5 mm in diameter and is often associated with visible scarring [3].

For patients with moderate to severe acne who are unresponsive to antibiotic treatment, the main line of treatment is oral isotretinoin [4]. Oral isotretinoin, a derivative of vitamin A, decreases the amount of oil released from the sebaceous glands and has anti-inflammatory effects. Moreover, isotretinoin reduces the presence of Cutibacterium acnes bacteria in the skin [5]. The efficacy of isotretinoin is well-established, yet it has various documented physical side effects which limit its usage. Furthermore, isotretinoin has been postulated to have a psychological impact, ranging from mood changes to suicidal ideation. These associations have been suggested by numerous studies, but causality has yet to be determined.

Many studies have explored the association between isotretinoin usage and psychiatric comorbidities, although, none of these studies have established a positive link [6].

In Saudi Arabia, there has been no research conducted to explore the psychological impact of isotretinoin usage on patients suffering from acne. The objective of this study was to define the impact of isotretinoin therapy and its physical side effects on the psychological status of acne patients and determine whether there is any improvement or worsening of the patients' mental status during treatment. This article was presented as an abstract in the AtlanticDerm, Montréal conference during its preliminary stages as an abstract on April 9-11, 2021.

## Materials and methods

This study was a cross-sectional survey conducted at the dermatology outpatient clinic at King Abdulaziz Medical City, Jeddah, Saudi Arabia. The sample size was calculated using the Raosoft sample size calculator [Raosoft (2015) Raosoft Sample Size Calculator. Raosoft, Inc., Seattle] which set the sample size to be 80 participants to maintain a confidence interval of 95% and a margin of error of 5%. Therefore, to reach the sample size calculated, patients diagnosed with acne vulgaris between January and April 2020 were included in the study. The study included 80 patients with acne vulgaris who were currently or previously treated with isotretinoin. Patients who were lost to follow-up, not compliant with the medication, or did not receive the medication (prescribed but not yet dispensed) were excluded. Ethical approval was obtained from the International Review Board of King Abdullah International Medical Research Center (KAIMRC).

We collected demographic and baseline data for each patient, including their gender, age, the number of years the patient had acne before using isotretinoin, the duration and dosage of treatment, and the level of adherence to the doctor’s recommendations. The participants were asked if they had any previous diagnosis of mental disorders, such as depression, generalized anxiety, or bipolar disorder. In addition, the commonly reported side effects of isotretinoin therapy were evaluated for each of the study subjects.

The participants were assessed using the Depression and Anxiety Stress Scale-21 (DASS-21), which is a self-reporting scale designed by Lovibond and Lovibond to measure the emotional states of depression, anxiety, and stress [7,8]. The questionnaire had a high reliability, with a Cronbach’s alpha value of 0.8 for depression, 0.89 for anxiety, and 0.78 for stress [7]. The questionnaire (available for use in research with no prior permission required - as indicated on their official site), contains three sets of sub-scales, with each sub-scale focusing on a different disease. Each scale contains seven questions that evaluate the key symptoms of each disease. The questions are scored on a scale of 0 to three, with 0 translating to “did not apply to me at all” and three translating to “applied to me most of the time”. The final score was calculated by multiplying the score of each set by two. When calculated as a sum, the final scores correspond to a severity in one of the following categories: normal, mild, moderate, severe, or extremely severe for each disease. Because Arabic is the native language of most of the patients at our hospital, we used a validated Arabic version of the DASS-21.

The descriptive statistics are presented as counts, proportions (%), means, and standard deviations, as appropriate. The Fischer exact test was used to establish the relationships between the levels of depression, anxiety, and stress and the baseline demographic characteristics of the acne patients using isotretinoin. A multivariate regression analysis was also conducted to determine the presence of any significant independent factors associated with depression, anxiety, and stress. A P-value of ≤0.05 was considered statistically significant. All the data analyses were performed using the Statistical Package for Social Sciences, version 21 (SPSS, Armonk, NY, USA).

## Results

As described in Table 1 below, 26-35-year-olds represented the most common age group in the study (33.8%), and the majority of the patients were female (81.3%). Furthermore, nearly half of the participants (45%) had acne for two to five years prior to treatment. Most of the respondents used isotretinoin to treat their acne for more than two months (72.5%). When asked if they adhered to their doctor’s recommendations, 71.5% of the patients stated, “most of the time”. The most common isotretinoin dosage reported to have been used by the respondents was 40 mg (27.5%). Notably, 7.6% of the patients documented a history of mental illness, including 3.8% who reported a previous history of major depressive disorders, 2.5% who reported having bipolar disorder, and 1.3% who reported generalized anxiety disorder.

**Table 1:** Basic demographic characteristics and isotretinoin usage in participants (n=80)

Study variables	n (%)
Age group	
<18 years	1 (1.3%)
18 – 21 years	19 (23.8%)
22 – 25 years	25 (31.3%)
26 – 35 years	27 (33.8%)
36 – 45 years	8 (10.0%)
Gender	
Male	15 (18.8%)
Female	65 (81.3%)
Years of acne vulgaris	
≤2 years	20 (25.0%)
2 – 5 years	36 (45.0%)
6 – 10 years	18 (22.5%)
>10 years	06 (07.5%)
Currently using Isotretinoin	
Yes	16 (20.0%)
No	64 (80.0%)
Duration of isotretinoin usage	
≤2 months	12 (15.0%)
>2 months	58 (72.5%)
Currently using	10 (12.5%)
Adherence to doctor recommendation	
Did not apply to me at all	3 (3.8%)
Applied to some degree	09 (11.3%)
Applied to considerable degree	11 (13.8%)
Applied most of the time	57 (71.3%)
Dose of Isotretinoin	
10 mg	3 (3.8%)
20 mg	18 (22.5%)
30 mg	19 (23.8%)
40 mg	22 (27.5%)
>40 mg	08 (10.0%)
I don’t know	10 (12.5%)
Frequency of moisturizer used	
Once per day	20 (25.0%)
Twice per day	28 (35.0%)
3 times per day	21 (26.3%)
4 times per day	11 (13.8%)
Previous history of mental disorder	
Major depressive disorder	3 (3.8%)
Generalized anxiety disorder	1 (1.3%)
Both depression and anxiety	2 (2.5%)
Bipolar disorder	2 (2.5%)
None of the above	72 (90.0%)

**Figure 1:**
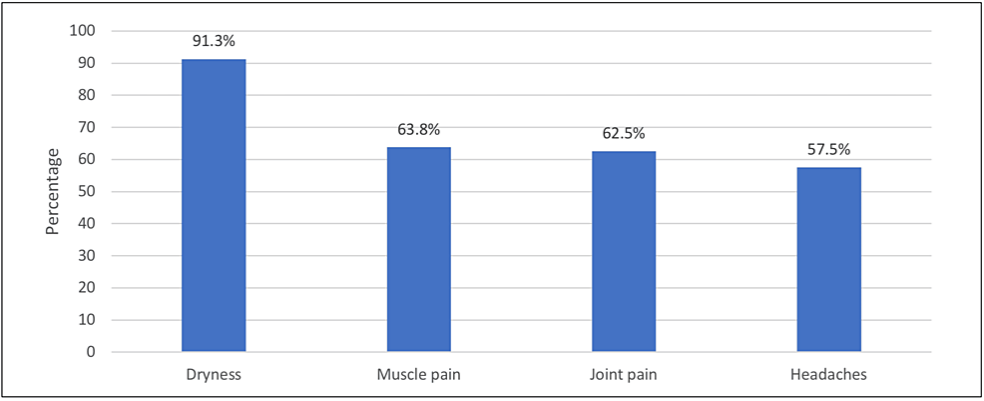
Side effects experienced by the isotretinoin user

As shown in Figure 1, the most common side effect reported by the respondents was dryness (91.3%). This was followed by muscle pain (63.8%), joint pain (62.5%), and headaches (57.5%).

Table 2 shows the descriptive statistics recorded for depression, anxiety, and stress in accordance with the DASS-21 assessment. The assessment identified depression among 20% of the study population [mean 4.95; standard deviation (SD) 7.03] while the rest of the patients (80%) exhibited no signs of depression. Furthermore, anxiety was observed in 27.5% of the patients (mean, 4.97; SD, 5.94), and approximately 16.3% of the respondents reported experiencing the perception of excessive stress (mean 7.25; SD 8.08). 

**Table 2:** Descriptive statistical numerics of depression, anxiety, and stress based on DASS-21

Variables	n (%)
Depression (mean ± SD)	4.95 ± 7.03
Depressed	16 (20.0%)
Not depressed	64 (80.0%)
Anxiety (mean ± SD)	4.97 ± 5.94
Anxious	22 (27.5%)
Not anxious	58 (72.5%)
Stress (mean ± SD)	7.25 ± 8.08
Stressed	13 (16.3%)
Not stressed	67 (83.8%)

**Figure 2:**
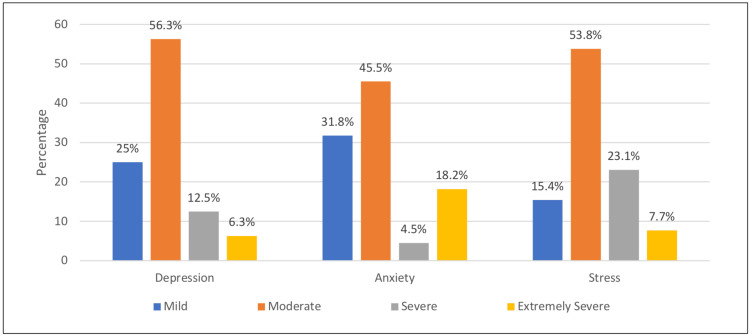
Severity of depression, anxiety, and stress

Figure 2 depicts the severity levels of depression, anxiety, and stress among the patients based on the DASS-21 criteria. Of those who were classified as depressed, 56.3% were moderately depressed, while 6.3% had extremely severe depression. Of the respondents who were anxious, 45.5% were classified as moderately anxious, 4.5% were severely anxious, and 18.2% had extremely severe anxiety. Among the patients who reported stress, 53.8% were in the moderate range, while 7.7% categorized their stress as extremely severe.

The Fischer exact test was used to analyze the relationships between depression, anxiety, stress and the usage of isotretinoin among the respondents. Based on the results of the Fischer exact test (shown in Table 3) a history of mental disorders showed a significant relationship with depression (X2=16.806; p=0.001). None of the other patient characteristics (age, sex, years with acne vulgaris, and duration and dosage of therapy) showed significant relationships with depression, anxiety, or stress (p-value >0.05).

**Table 3:** Relationship between depression, anxiety, and stress in reference to demographic characteristics and isotretinoin usage (n=80) §P-value has been calculated using the Fischer exact test; ** Significant at p≤0.05 level.

Factor	Depression		Anxiety		Stress	
	Yes n (%) ^(n=16)^	No n (%) ^(n=64)^	Yes n (%) ^(n=22)^	No n (%) ^(n=58)^	Yes n (%) ^(n=13)^	No n (%) ^(n=67)^
Age group						
≤25 years	13 (81.3%)	58 (92.1%)	18 (85.7%)	53 (91.4%)	10 (76.9%)	61 (92.4%)
>25 years	03 (18.8%)	05 (07.9%)	03 (14.3%)	05 (08.6%)	03 (23.1%)	05 (07.6%)
X2; P-value	1.639; 0.348		0.544; 0.432		2.867; 0.120	
Gender						
Male	01 (06.3%)	14 (21.9%)	02 (09.1%)	13 (22.4%)	01 (07.7%)	14 (20.9%)
Female	15 (93.8%)	50 (78.1%)	20 (90.9%)	45 (77.6%)	12 (92.3%)	53 (79.1%)
X2; P-value	2.051; 0.281		1.858; 0.215		1.246; 0.443	
Years of Acne vulgaris						
≤5 years	13 (81.3%)	43 (67.2%)	17 (77.3%)	39 (67.2%)	10 (76.9%)	46 (68.7%)
>5 years	03 (18.8%)	21 (32.8%)	05 (22.7%)	19 (32.8%)	03 (23.1%)	21 (31.3%)
X2; P-value	1.205; 0.367		0.764; 0.428		0.354; 0.745	
Currently using Isotretinoin						
Yes	05 (31.3%)	11 (17.2%)	04 (18.2%)	12 (20.7%)	02 (15.4%)	14 (20.9%)
No	11 (68.8%)	53 (82.8%)	18 (81.8%)	46 (79.3%)	11 (84.6%)	53 (79.1%)
X2; P-value	1.582; 0.292		0.063; 1.000		0.207; 1.000	
Duration of Isotretinoin used						
≤2 months	04 (25.0%)	08 (12.5%)	04 (18.2%)	08 (13.8%)	01 (07.7%)	11 (16.4%)
>2 months	09 (56.3%)	49 (76.6%)	15 (68.2%)	43 (74.1%)	10 (76.9%)	48 (71.6%)
Currently using	03 (18.8%)	07 (10.9%)	03 (13.6%)	07 (12.1%)	02 (15.4%)	08 (11.9%)
X2; P-value	2.687; 0.309		0.314; 0.784		0.698; 0.790	
Adherence to doctor’s advised						
Did not apply to me at all	0	03 (04.7%)	01 (04.5%)	02 (03.4%)	0	03 (04.5%)
Applied to some degree	01 (06.3%)	08 (12.5%)	03 (13.6%)	06 (10.3%)	01 (07.7%)	08 (11.9%)
Applied to great degree	01 (06.3%)	10 (15.6%)	03 (13.6%)	08 (13.8%)	03 (23.1%)	08 (11.9%)
Applied most of the time	14 (87.5%)	43 (67.2%)	15 (68.2%)	42 (72.4%)	09 (69.2%)	48 (71.6%)
X2; P-value	2.754; 0.458		0.245; 0.962		1.748; 0.763	
Dose of Isotretinoin						
10 mg	0	03 (04.7%)	0	03 (05.2%)	0	03 (04.5%)
20 mg	05 (31.3%)	13 (20.3%)	05 (22.7%)	13 (22.4%)	03 (23.1%)	15 (22.4%)
30 mg	02 (12.5%)	17 (26.6%)	05 (22.7%)	14 (24.1%)	01 (07.7%)	18 (26.9%)
40 mg	06 (37.5%)	16 (25.0%)	08 (36.4%)	14 (24.1%)	07 (53.8%)	15 (22.4%)
>40 mg	01 (06.3%)	07 (10.9%)	03 (13.6%)	05 (08.6%)	01 (07.7%)	07 (10.4%)
I don’t know	02 (12.5%)	08 (12.5%)	01 (04.5%)	09 (15.5%)	01 (07.7%)	09 (13.4%)
X2; P-value	3.505; 0.718		3.956; 0.637		6.557; 0.319	
Frequency of moisturizer used						
Once per day	05 (31.3%)	15 (23.4%)	04 (18.2%)	16 (27.6%)	04 (30.8%)	16 (23.9%)
Twice per day	04 (25.0%)	24 (37.5%)	06 (27.3%)	22 (37.9%)	02 (15.4%)	26 (38.8%)
3 times per day	03 (18.8%)	18 (28.1%)	06 (27.3%)	15 (25.9%)	04 (30.8%)	17 (25.4%)
4 times per day	04 (25.0%)	07 (10.9%)	06 (27.3%)	05 (08.6%)	03 (23.1%)	08 (11.9%)
X2; P-value	3.153; 0.381		5.130; 0.196		3.016; 0.344	
History of mental disorder						
Yes	06 (37.5%)	02 (03.1%)	04 (18.2%)	04 (06.9%)	02 (15.4%)	06 (09.0%)
No	10 (62.5%)	62 (96.9%)	18 (81.8%)	54 (93.1%)	11 (84.6%)	61 (91.0%)
X2; P-value	16.806; 0.001 **		2.257; 0.206		0.500; 0.610	

As shown in Table 4, we observed that the presence of muscle pain showed a significant relationship with anxiety (X2=4.287; p=0.042). Additionally, the presence of joint pain exhibited a positive relationship with depression (X2=12.000; p<0.001), anxiety (X2=10.449; p=0.002), and stress (X2=5.884; p=0.025). Headaches were also observed to have a positive relationship with depression (X2=7.366; p=0.010) and anxiety (X2=4.855; p=0.042), but not with stress (X2=0.874; p=0.541). Conversely, dryness did not show a significant relationship with depression, anxiety, or stress (all p>0.05).

**Table 4:** Relationship between the side effects experienced by the participants in relation to depression, anxiety, and stress (n=80) §P-value has been calculated using the Fischer exact test; ** Significant at p≤0.05 level.

Factor	Depression		Anxiety		Stress	
	Yes n (%) ^(n=16)^	No n (%) ^(n=64)^	Yes n (%) ^(n=22)^	No n (%) ^(n=58)^	Yes n (%) ^(n=13)^	No n (%) ^(n=67)^
Muscle pain						
Yes	13 (81.3%)	38 (59.4%)	18 (81.8%)	33 (56.9%)	11 (84.6%)	40 (59.7%)
No	03 (18.8%)	26 (40.6%)	04 (18.2%)	25 (43.1%)	02 (15.4%)	27 (40.3%)
X2; P-value	2.650; 0.148		4.287; 0.042 **		2.924; 0.119	
Joint pain						
Yes	16 (100%)	34 (53.1%)	20 (90.9%)	30 (51.7%)	12 (92.3%)	38 (56.7%)
No	0	30 (46.9%)	02 (09.1%)	28 (48.3%)	01 (07.7%)	29 (43.3%)
X2; P-value	12.000; <0.001 **		10.449; 0.002 **		5.884; 0.025 **	
Headaches						
Yes	14 (87.5%)	32 (50.0%)	17 (77.3%)	29 (50.0%)	09 (69.2%)	37 (55.2%)
No	02 (12.5%)	32 (50.0%)	05 (22.7%)	29 (50.0%)	04 (30.8%)	30 (44.8%)
X2; P-value	7.366; 0.010 **		4.855; 0.042 **		0.874; 0.541	
Dryness						
Yes	14 (87.5%)	59 (92.2%)	20 (90.9%)	53 (91.4%)	12 (92.3%)	61 (91.0%)
No	02 (12.5%)	05 (07.8%)	02 (09.1%)	05 (08.6%)	01 (07.7%)	06 (09.0%)
X2; P-value	0.352; 0.622		0.004 ; 1.000		0.022; 1.000	

The multivariate regression analysis (Table 5) revealed that respondents with a history of mental disorders were 38.2 times more likely to develop depression [adjusted odds ratio (AOR)=38.2; 95% confidence interval (CI)=2.5. 582; p=0.009]. Moreover, respondents with joint pain were 6.9 times more likely to develop anxiety (AOR=6.9; 95% CI=1.0. 48.5; p=0.050). Headaches, however, did not show a significant relationship with depression, anxiety, or stress (p>0.05).

**Table 5:** Multivariate regression analysis to determine the independent significant factor associated with depression, anxiety, and stress (n=80) AR: adjusted odds ratio, ** Significant at p≤0.05 level.

Factor	Depression		Anxiety		Stress	
	AOR (95% CI)	P-value	AOR (95% CI)	P-value	AOR (95% CI)	P-value
History of mental disorder						
Yes	38.2 (2.5 – 582)	0.009**	2.2 (0.4 – 10.5)	0.339	1.3 (0.2 – 7.46)	0.794
No	Ref		Ref		Ref	
Muscle pain						
Yes	0.26 (0.3 – 2.2)	0.216	0.9 (0.2 – 4.6)	0.940	1.2 (0.2 – 8.21)	0.825
No	Ref		Ref		Ref	
Joint pain						
Yes	--	--	6.9 (1.0 – 48.5)	0.050**	8.6 (0.7 – 105)	0.091
No	Ref		Ref		Ref	
Headaches						
Yes	6.9 (0.7 – 70)	0.103	1.7 (0.5 – 6.17)	0.398	0.8 (0.2 – 3.37)	0.775
No	Ref		Ref		Ref	

## Discussion

Isotretinoin has been established as one of the most efficacious treatments in use for the management of moderate to severe acne vulgaris. The synthetic retinoid has many documented side effects, including xerosis, facial erythema, epistaxis, muscle aches, pruritus, fatigue, and joint pain. Among the subjects in the current study, the most prevalent adverse effect documented was dryness, followed by muscle pain, joint pain, and headaches. Our results are in agreement with a five-year observational study carried out in Poland and Romania, where dry lips and xerosis of the skin were also the most prevalent consequences of isotretinoin therapy [9,10].

In 1983, the first study reporting depression as a possible side effect of isotretinoin therapy was published [11]. Subsequently, the United States Food and Drug Administration issued a warning about the potential correlation between isotretinoin use and adverse psychological effects, such as psychosis, depression, and suicidal ideation. However, many ensuing studies have failed to establish a correlation between isotretinoin therapy and any psychological impacts. Some studies have even inferred that isotretinoin improves the psychological status of acne patients due to the observation of improvements in the conditions of patients being treated with the medication [12,13]. In 2008, however, a case-crossover study showed a statistically significant association between isotretinoin therapy and depression. Thus, recommendations have been made to closely monitor patients whenever isotretinoin is prescribed [14].

In our study, most of the subjects enrolled were female. The primary age group consisted of patients aged 26-35 years. More than half of the study subjects used isotretinoin for two months or more. The DASS-21 assessment was employed as a screening tool to identify any psychological impacts associated with isotretinoin therapy. The DASS-21 assessment is a self-reporting instrument composed of 21 questions that measure depression, anxiety, and stress, and it displays the results interpreted as different categories of severity. Another study that aimed to identify the correlation between isotretinoin therapy and the psychological status of patients using the medication employed three questionnaires to serve their purpose (the Beck Depression Inventory, the State-Trait Anxiety Inventory, and the Measure of Psychological Stress screening tool) [15]. Their results indicated that there was no association between isotretinoin therapy and psychiatric morbidity. On the contrary, they observed an improved quality of life among their patients after therapy. Our results demonstrate that the predominant psychiatric disorder among the study patients taking isotretinoin was anxiety. In addition, depression was also significantly prevalent among the study population. Among the various factors evaluated, only a history of mental illness was statistically proven to be an independent risk factor for developing depression when isotretinoin was used. 

The adverse physical effects commonly reported by patients taking isotretinoin were evaluated to assess whether there is a significant and independent risk of the development of depression, anxiety, or stress. We observed that the presence of muscle pain as an adverse effect increased the likelihood of the patients developing anxiety. In addition, the patients who suffered from arthralgia had a significantly increased risk of developing depression, anxiety, and stress. Furthermore, respondents who reported headaches had a higher risk of perceiving anxiety or depression but interestingly, not stress. However, xerosis was not associated with any of the psychiatric morbidities evaluated by the DASS-21. 

To our knowledge, this is the first study to investigate correlations between the adverse effects commonly reported by patients using isotretinoin and the impact each adverse effect has on the patients’ psychological status. One of the limitations encountered in this study was the small sample size due to the inclusion of only patients who were on isotretinoin therapy for a minimum duration of one month. Another limitation was the study’s cross-sectional survey design. Additionally, the study population consisted primarily of females. The predominance of females in the study could be attributed to their increased cosmetic concern and heightened awareness of appearance, compared to males [16,17].

## Conclusions

Isotretinoin therapy is a highly potent acne treatment. Although it has proven to be successful in the management of acne vulgaris, neglecting its psychiatric impact on users can lead to non-compliance and further disease deterioration. Based on our results, we recommend that isotretinoin be used with extreme caution when a patient has a history of mental illness. Additionally, the presence of any treatment side effects should be evaluated and promptly managed, as they have been shown to increase the risk of deteriorating the patients’ psychological status. Further studies with larger populations are required, however, to accurately delineate the psychological impact that isotretinoin imposes on patients. Overall, the physical side effect profile is well acknowledged, yet the exact psychological impact that isotretinoin predisposes patients to is yet to be determined.
